# Supplementary material for: The chloroplast genome inheritance pattern of the Deli-Nigerian prospection material (NPM) × Yangambi population of Elaeis guineensis Jacq
Source: PeerJ. 2024 May 27;12:e17335. doi: 10.7717/peerj.17335 (PMC11138521; doi:10.7717/peerj.17335)
Supplement: Table S1 [file peerj-12-17335-s001.docx]

| Table S1. Raw reads sequencing details. | | | |  |  |
| --- | --- | --- | --- | --- | --- |
| Sample | **SRA Accession** | **Total_Reads** | **Total_Bases** | **Q30%** | **GC%** |
| ML-161 | SRR24011240 | 398341772 | 59677199365 | 96.33 | 37.33 |
| J4-25 | SRR24011239 | 309786730 | 46400399601 | 95.95 | 37.48 |
| GB33-1 | SRR24011228 | 208352192 | 31208458969 | 95.68 | 37.39 |
| GB33-2 | SRR24011222 | 194470736 | 29141328777 | 96.89 | 38.11 |
| GB33-3 | SRR24011221 | 206560238 | 30942194233 | 95.68 | 36.94 |
| GB33-4 | SRR24011220 | 168223126 | 25200839866 | 96 | 37.97 |
| GB33-5 | SRR24011219 | 221622524 | 33193485895 | 95.45 | 37.61 |
| GB33-6 | SRR24011218 | 165601602 | 24772911982 | 91.6 | 39.1 |
| GB33-9 | SRR24011217 | 126179838 | 18903632149 | 95.26 | 37.29 |
| GB33-11 | SRR24011216 | 204660264 | 30666621980 | 96.52 | 38.58 |
| GB33-13 | SRR24011238 | 226644788 | 33955025144 | 96.41 | 37.39 |
| GB33-17 | SRR24011237 | 191873954 | 28742279212 | 96.01 | 37.68 |
| GB33-19 | SRR24011236 | 189242908 | 28341224918 | 95.09 | 37.53 |
| GB33-26 | SRR24011235 | 244121762 | 36570135166 | 96.28 | 37.91 |
| GB33-27 | SRR24011234 | 216373546 | 32417495261 | 96.34 | 38.14 |
| GB33-29 | SRR24011233 | 182560024 | 27349266781 | 96.04 | 38.16 |
| GB33-31 | SRR24011232 | 194356614 | 29112280217 | 95.74 | 38.05 |
| GB33-32 | SRR24011231 | 230877212 | 34586059496 | 95.99 | 38.3 |
| GB33-40 | SRR24011230 | 197095380 | 29530690077 | 96.37 | 38.06 |
| GB33-41 | SRR24011229 | 209999232 | 31459056723 | 96.14 | 38.21 |
| GB33-44 | SRR24011227 | 221728620 | 33215481340 | 96.09 | 37.22 |
| GB33-46 | SRR24011226 | 159272960 | 23709016693 | 93.06 | 37.51 |
| GB33-47 | SRR24011225 | 143216406 | 21426994862 | 91.94 | 38.81 |
| GB33-48 | SRR24011224 | 214141600 | 32051554047 | 93.14 | 37.2 |
| GB33-51 | SRR24011223 | 221622524 | 33193485895 | 95.45 | 37.61 |
