# Supplementary material for: The chloroplast genome inheritance pattern of the Deli-Nigerian prospection material (NPM) × Yangambi population of Elaeis guineensis Jacq
Source: PeerJ. 2024 May 27;12:e17335. doi: 10.7717/peerj.17335 (PMC11138521; doi:10.7717/peerj.17335)
Supplement: Table S2 — Information on average chloroplast genome coverage, chloroplast genome reads and chloroplast genome size among all 24 chloroplast genomes analysed in this study. [file peerj-12-17335-s002.docx]

| Table S2:  Chloroplast genome feature of 24 *Elaeis guineensis* individuals in this study | | | | | |
| --- | --- | --- | --- | --- | --- |
| No. | **Sample Individuals** | **Average cp genome coverage** **(X)** | **Cp genome reads** | **Genome size (bp)** | **Accession number in Genbank** |
| 1. | ML-161 | 4,783 | 760,541 | 156,988 | OR125032 |
| 2. | J4-25 | 1,500 | 547,017 | 156,983 | OR120533 |
| 3. | GB331 | 1,270 | 1,198,366 | 156,983 | OR125056 |
| 4. | GB332 | 829 | 1,199,956 | 156,983 | OR125055 |
| 5. | GB333 | 1,258 | 586,232 | 156,983 | OR125054 |
| 6. | GB334 | 857 | 1,513,654 | 156,983 | OR125053 |
| 7. | GB335 | 1,211 | 1,079,430 | 156,983 | OR125052 |
| 8. | GB336 | 1,504 | 1,584,768 | 156,983 | OR125051 |
| 9. | GB339 | 613 | 693,562 | 156,983 | OR125050 |
| 10. | GB3311 | 961 | 292,146 | 156,983 | OR125049 |
| 11. | GB3313 | 1,648 | 1,751,710 | 156,983 | OR125048 |
| 12. | GB3317 | 1,360 | 1,840,410 | 156,982 | OR125047 |
| 13. | GB3319 | 1,116 | 1,061,988 | 156,983 | OR125046 |
| 14. | GB3326 | 1,960 | 1,420,608 | 156,983 | OR125045 |
| 15. | GB3327 | 1,914 | 1,535,656 | 156,983 | OR125044 |
| 16. | GB3329 | 1,585 | 1,693,908 | 156,983 | OR125043 |
| 17. | GB3331 | 2,083 | 1,854,666 | 156,983 | OR125042 |
| 18. | GB3332 | 2,084 | 1,654,898 | 156,983 | OR125041 |
| 19. | GB3340 | 1,290 | 1,236,842 | 156,983 | OR125040 |
| 20. | GB3341 | 1,016 | 832,324 | 156,984 | OR125039 |
| 21. | GB3344 | 1,867 | 1,534,010 | 156,983 | OR125038 |
| 22. | GB3347 | 1,054 | 324,972 | 156,983 | OR125036 |
| 23. | GB3348 | 1,753 | 1,241,270 | 156,983 | OR125035 |
| 24. | GB3351 | 1,459 | 1,087,428 | 156,983 | OR125034 |
