# Supplementary material for: The chloroplast genome inheritance pattern of the Deli-Nigerian prospection material (NPM) × Yangambi population of Elaeis guineensis Jacq
Source: PeerJ. 2024 May 27;12:e17335. doi: 10.7717/peerj.17335 (PMC11138521; doi:10.7717/peerj.17335)
Supplement: Table S3 — Details on the nucleotide variations present among all chloroplast genomes analyzed in this study. [file peerj-12-17335-s003.docx]

| Table S3:  Nucleotide variation in all samples in this study by comparison to maternal cp genome (J425). | | | | | | | | | |
| --- | --- | --- | --- | --- | --- | --- | --- | --- | --- |
| Variation Type | **Region/Locus** | **Alignment Position (bp)** | ***Elaeis guineensis* individuals** | | | | | | |
|  |  |  | 1 | 2 | 3 | 4 | 5 | 6 | 7 |
| SNP | LSC / *ndh*C - *trn*V-UAC (intergenic) | 10871 | A | **G** | A | A | A | A | **G** |
| SNP | IR / *rrn*23 (coding) | 107148 | A | A | **T** | A | A | A | A |
| Insertion | LSC / *paf*ll-*cem*A (intergenic) | 6902 | - | - | - | - | **C** | - | - |
| Deletion | LSC / *rpl*16 (intron) | 83823 | T | T | T | **-** | T | T | T |
| Insertion | IR / *rrn*23 (coding) | 107147 | - | - | - | - | - | **T** | - |
| SNP | SSC / *ndh*F (coding) | 113667 | **A** | C | C | C | C | C | C |
| SNP | LSC / *atp*F (intron) | 13146 | **A** | G | G | G | G | G | G |
| SNP | LSC / *rps*3 (coding) | 84326 | **A** | G | G | G | G | G | G |
| SNP | IR / *rpl*2 (exon) | 87116 | **A** | G | G | G | G | G | G |
| SNP | SSC / *ndh*D-*psa*C (intergenic) | 118629 | **A** | T | T | T | T | T | T |
| SNP | SSC / *ndh*D-*psa*C (intergenic) | 118638 | **A** | T | T | T | T | T | T |
| SNP | SSC / *ccs*A (coding) | 116592 | **C** | T | T | T | T | T | T |
| SNP | LSC / *psb*M-*trn*D-GUC (intergenic) | 30388 | **G** | T | T | T | T | T | T |
| SNP | LSC / *trn*V-UAC – *trn*M-CAU (intergenic) | 53627 | **G** | T | T | T | T | T | T |
| SNP | SSC / *ndh*D-*psa*C (intergenic) | 118627 | **T** | A | A | A | A | A | A |
| SNP | SSC / *ndh*D-*psa*C (intergenic) | 118633 | **T** | A | A | A | A | A | A |
| SNP | SSC / *ndh*D-*psa*C (intergenic) | 118634 | **T** | A | A | A | A | A | A |
| SNP | SSC / *ndh*D-*psa*C (intergenic) | 118640 | **T** | A | A | A | A | A | A |
| SNP | LSC / *rpl*16 (intron) | 83655 | **T** | C | C | C | C | C | C |
| SNP | IR / *rpl*2 (exon) | 155076 | **T** | C | C | C | C | C | C |
| Deletion | LSC / *atp*F (intron) | 12995 | **-** | T | T | T | T | T | T |
| Deletion | LSC / *trn*D-GUC – *trn*Y-GUA (intergenic) | 31274 | **-** | T | T | T | T | T | T |
| Insertion | LSC / *trn*S-GCU – *trn*G-UCC (intergenic) | 9209 | **A** | - | - | - | - | - | - |
| Insertion | LSC / *acc*D-*psa*I (intergenic) | 60560 | **A** | - | - | - | - | - | - |
| Insertion | IR / *rps*19-*psb*A (intergenic) | 156938 | **A** | - | - | - | - | - | - |
| Insertion | LSC / *clp*P1 (intron) | 71490 | **T** | - | - | - | - | - | - |
| Insertion | LSC / *clp*P1 (intron) | 72191 | **T** | - | - | - | - | - | - |
| Insertion | IR / *rpl*22-*rps*19 (intergenic) | 85247 | **T** | - | - | - | - | - | - |
| Insertion | SSC / *rpl*32 – *trn*L-UAG (intergenic) | 115098 | **T** | - | - | - | - | - | - |

*1-ML161, 2-GB331, 3-GB3311, 4-GB3317, 5-GB3341, 6-GB3346, 7-GB3347
