# Supplementary material for: The chloroplast genome inheritance pattern of the Deli-Nigerian prospection material (NPM) × Yangambi population of Elaeis guineensis Jacq
Source: PeerJ. 2024 May 27;12:e17335. doi: 10.7717/peerj.17335 (PMC11138521; doi:10.7717/peerj.17335)
Supplement: Table S4 — Locations of SNPs and InDels found in the chloroplast genome among all samples used in this study. [file peerj-12-17335-s004.docx]

| Table S4:  Variations (SNPs and InDels) detected among chloroplast genome sequences compared to J4-25 chloroplast genome | | | | | | | |
| --- | --- | --- | --- | --- | --- | --- | --- |
| No | **Sample Individuals** | **Alignment position** | **Var. type** | **Nucleotide variations** | **Location in the cp genome / gene** | **Original position in the cp genome** | **Coverage depth (X) in nucleotide position** |
| 1. | ML-161 | 113667 | SNP | 'A' instead of 'C' | SSC- ndhF (coding gene) | 113655 | 3543 |
| 2. | ML-161 | 13146 | SNP | 'A' instead of 'G' | LSC- atpF (intron) | 13144 | 4364 |
| 3. | ML-161 | 84332 | SNP | 'A' instead of 'G' | LSC- rps3 (coding gene) | 84323 | 5438 |
| 4. | ML-161 | 87123 | SNP | 'A' instead of 'G' | IR- rpl2 (exon) | 87113 | 5197 |
| 5. | ML161 | 118637 | SNP | 'A' instead of 'T' | SSC- ndhD-psaC (intergenic) | 118625 | 4209 |
| 6. | ML161 | 118646 | SNP | 'A' instead of 'T' | SSC- ndhD-psaC (intergenic) | 118634 | 4149 |
| 7. | ML161 | 116600 | SNP | 'C' instead of 'T' | SSC- ccsA (coding gene) | 116588 | 4185 |
| 8. | ML161 | 30389 | SNP | 'G' instead of 'T' | LSC- psbM-trnD-GUC (intergenic) | 30386 | 5416 |
| 9. | ML161 | 53628 | SNP | 'G' instead of 'T' | LSC- trnV-UAC-trnM-CAU (intergenic) | 53624 | 4990 |
| 10. | ML161 | 118635 | SNP | 'T' instead of 'A' | SSC- ndhD-psaC (intergenic) | 118623 | 4224 |
| 11. | ML161 | 118641 | SNP | 'T' instead of 'A' | SSC- ndhD-psaC (intergenic) | 118629 | 4211 |
| 12. | ML161 | 118642 | SNP | 'T' instead of 'A' | SSC- ndhD-psaC (intergenic) | 118630 | 4198 |
| 13. | ML161 | 118648 | SNP | 'T' instead of 'A' | SSC- ndhD-psaC (intergenic) | 118636 | 3806 |
| 14. | ML161 | 83660 | SNP | 'T' instead of 'C' | LSC- rpl16 (intron) | 83652 | 4783 |
| 15. | ML161 | 155086 | SNP | 'T' instead of 'C' | IR- rpl2 (exon) | 155072 | 5293 |
| 16. | ML161 | 12995 | InDel | Deletion 'T' | LSC- atpF (intron-coding gene) | NA | NA |
| 17. | ML161 | 31275 | InDel | Deletion 'T' | LSC - trnD-GUC - trnY-GUA (intergenic) | NA | NA |
| 18. | ML161 | 9212 | InDel | Insertion 'A' | LSC- trnS-GCU – trnG-UCC (intergenic) | 9211 | 4959 |
| 19. | ML161 | 60561 | InDel | Insertion 'A' | LSC- accD-psaI (intergenic) | 60557 | 3794 |
| 20. | ML161 | 156953 | InDel | Insertion 'A' | IR - rps19-psbA (intergenic) | 156939 | 2171 |
| 21. | ML161 | 71494 | InDel | Insertion 'T' | LSC- clpP1 (intron) | 71490 | 5103 |
| 22. | ML161 | 72198 | InDel | Insertion 'T' | LSC- clpP1 (intron) | 72194 | 5093 |
| 23. | ML161 | 85256 | InDel | Insertion 'T' | IR- rpl22-rps19 (intergenic) | 85246 | 4694 |
| 24. | ML161 | 115106 | InDel | Insertion 'T' | SSC- rpl32-trnL-UAG (intergenic) | 115094 | 2665 |
| 25. | GB3347 | 10871 | SNP | 'G' instead of 'A' | LSC- ndhC-trnV-UAC (intergenic) | 10869 | 1017 |
| 26. | GB3331 | 10871 | SNP | 'G' instead of 'A' | LSC- ndhC-trnV-UAC (intergenic) | 10869 | 2025 |
| 27. | GB3311 | 107156 | SNP | 'T' instead of 'A' | IR- rrn23 | 107141 | 409 |
| 28. | GB3341 | 6902 | InDel | Insertion 'C' | LSC- pafII-cemA (intergenic) | 6902 | 341 |
| 29. | GB3317 | 83828 | InDel | Deletion 'T' | LSC- rpl16 (intron) | NA | NA |
| 30. | GB3346 | 107155 | InDel | Insertion ‘T’ | IR – rrn23 | 107141 | 511 |
